# Patterns and risk factors of double burden of malnutrition among adolescent girls and boys in Indonesia

**DOI:** 10.1371/journal.pone.0221273

**Published:** 2019-08-20

**Authors:** Masumi Maehara, Jee Hyun Rah, Airin Roshita, Julia Suryantan, Asrinisa Rachmadewi, Doddy Izwardy

**Affiliations:** 1 Nutrition Unit, Child Survival and Development Cluster, United Nations Children’s Fund, Jakarta, Indonesia; 2 Savica Public Health & Communication Consultancy, Jakarta, Indonesia; 3 Directorate of Community Nutrition, Ministry of Health, Jakarta, Indonesia; Addis Ababa University School of Public Health, ETHIOPIA

## Abstract

**Objectives:**

As an emerging middle-income country, Indonesia is grappling with the double burden of malnutrition across all age groups, including adolescents. Slow gains in reducing undernutrition are compounded by rapidly increasing overnutrition. This study aims to determine the patterns and determinants of this double burden, particularly stunting, thinness and overweight, among adolescent girls and boys aged 12–18 years in Indonesia.

**Methods:**

A cross-sectional survey was conducted among 2,160 girls and boys in the districts of Klaten and Lombok Barat in 2017. Data were collected on adolescents’ nutritional status, sociodemographic characteristics, morbidity, dietary intake and physical activity and other relevant factors. Multivariable logistic regression models using generalized estimation equation were employed to determine risk factors for stunting, thinness and overweight.

**Results:**

About a quarter of adolescent girls (25%) and boys (21%) were stunted. Approximately 5% of girls and 11% of boys were thin, whereas 11% girls and boys each were overweight. Living in a higher wealth household (OR = 0.67; 95% CI: 0.49–0.91), compared to a lower wealth household, and living in a district with higher socioeconomic status (OR = 0.63; 95% CI: 0.51–0.79) were associated with lower odds of being stunted. Adolescent education was also protective against stunting (OR = 0.52; 95% CI: 0.33–0.88). Older adolescents aged 15–18 years were more likely to be stunted (OR = 1.88; 95% CI: 1.42–2.46). Being a girl was associated with reduced odds of being thin (OR = 0.42; 95% CI: 0.30–0.58). Higher household wealth (OR = 1.93; 95% CI: 1.27–2.97) predicted being overweight, while maternal primary or some secondary education, compared to no or incomplete primary education, was protective against adolescent overweight (OR = 0.60; 95% CI: 0.40–0.90).

**Conclusion:**

Indonesian adolescent girls and boys face both extreme spectrums of malnutrition. Addressing the dual burden of malnutrition requires a multi-pronged approach, and urgent shift is warranted in nutrition policy and programmes targeting adolescents to effectively address the associated underlying determinants.

## Introduction

The double burden of malnutrition (DBM) has emerged as a worldwide concern, in which undernutrition and overnutrition coexist in the same populations, households and individuals [1]. As an emerging middle-income country, Indonesia is a prime example of the DBM across the life course and spanning all wealth quintiles [1,2]. Both stunting and wasting remain prevalent, affecting about 37% and 12% of children under 5 years of age, respectively, whereas more than a quarter of Indonesian adults are overweight or obese [3].

Despite its middle-income status, reduction in undernutrition has been slow, whereas changes in dietary intake and physical activity levels associated with globalization, industrialization and urbanization contribute to the obesity epidemic [4]. Key drivers of these changes include the ease and convenience of food consumption including increased availability of packaged food products, alongside increasingly sedentary occupations and lifestyles [5]. With non-communicable diseases (NCDs) already accounting for over 70% of deaths in Indonesia, addressing diet- and lifestyle-related risk factors of NCDs is a high priority [6].

Adolescence is a period of rapid growth and development, and is a critical period for the acquisition of health-related behaviors such as food preferences and physical activity [7–11]. These behaviors usually track throughout the life course [10,11] and may contribute to nutrition-related NCDs in adulthood [12]. In 2013, Indonesia’s National Basic Health Research Survey reported that approximately one-third of adolescents aged 13–18 years were stunted (girls: 29%; boys: 38%), 11% of adolescents aged 13–15 years were thin (girls: 9%; boys: 13%), while a further 11% of adolescents of the same age were overweight or obese (girls: 10%; boys: 12%) [3]. Evidence suggests that poor dietary habits and sedentary lifestyle are ubiquitous among Indonesian school-age children and adolescents [5,13]. Widespread malnutrition among children and adolescents shapes their ability to develop and perform to their full potential, and in turn adversely affects the national development trajectory [14].

While the global and national policy agenda has maintained a strong focus on undernutrition particularly during the first 1,000 days of life, little attention has been paid to the nutritional status of adolescents. Few policies and programmes target the DBM in this age group, such as the guideline on balanced nutrition [15] and school health programme [16]. This study aims to examine the patterns and determinants of the DBM, specifically stunting, thinness and overweight, among adolescents in Indonesia. We believe the findings will provide an important evidence base for future design and implementation of nutrition policies and programmes targeting to improve the nutritional status of adolescents in Indonesia.

## Materials and methods

### Study design and subjects

A cross-sectional household survey was conducted in the districts of Klaten and Lombok Barat in April and May 2017. Klaten is one of the most densely populated municipalities in Central Java Province, with a total population of 1.2 million in 2015 [17]. Lombok Barat is one of 10 municipalities in West Nusa Tenggara Province, with a total population of 654,892 [17]. Klaten’s population generally has higher socioeconomic status than Lombok Barat’s counterparts [18,19]. The two districts were selected in consultation with the national and local governments, considering a number of factors such as adolescent nutritional status, cultural context and infrastructure.

A sample size of 1080 was determined to be sufficient to detect a 7.5-percentage point difference in key (i.e., dietary and physical activity) behaviors between girls and boys and for adolescents between the two districts. A total sample size of 2160 was sufficient to detect a 5-percentage point difference in behaviors across the whole sample. A representative sample from across both districts included adolescent girls and boys aged 12–18 years and their parents/guardians. Exclusion criteria included the following: 1) refusing an interview or anthropometric measurement; 2) having any mental or physical limitations that would inhibit completing the interview and assessments; and 3) not residing in the selected village at the time of the survey.

**Fig 1:**
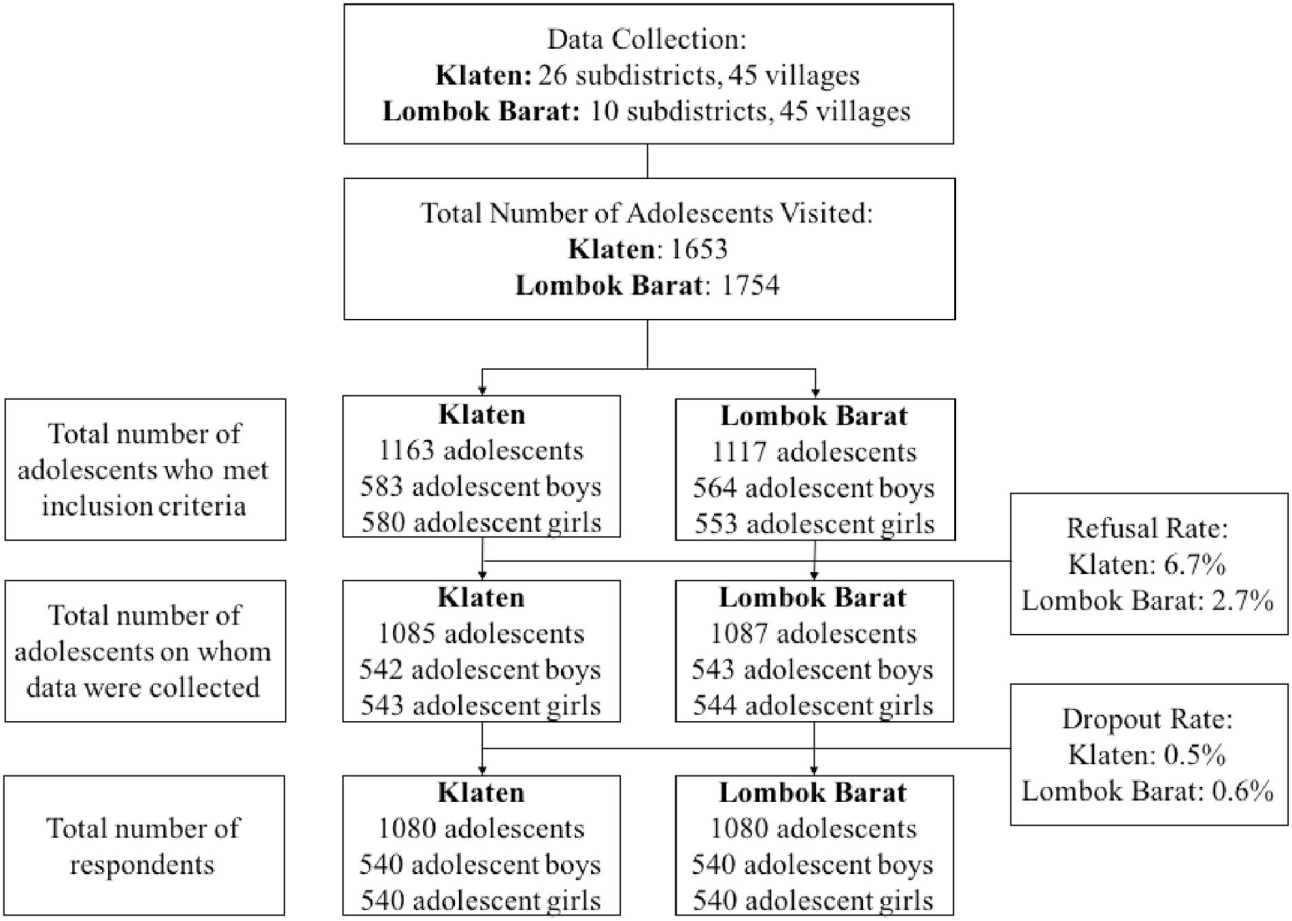
Respondents refusal and dropout rates in Klaten and Lombok Barat.

A multistage sampling procedure was employed. First, 45 clusters (villages) in each district were selected using a Probability Proportionate to Size sampling method. Second, one or more sub-villages were selected randomly to obtain at least 12 adolescent boys and 12 adolescent girls aged 12–18 years in each cluster. If there were more than 12 of either sex, we obtained 12 using simple random sampling. If there were less than 12 boys and 12 girls, a second and potentially a third sub-village in the same village were randomly selected until the required sample size was obtained (Fig 1). A total of 1,080 girls and 1,080 boys and 1,951 parents completed the survey. Only one adolescent from each household was included in the sample.

### Data collection

All selected eligible adolescents were visited for home-based interviews and assessments. Interviews were conducted either in Indonesian or the local language. To the extent possible, interviews were administered separately for adolescents and their parents/guardians in order to minimize potential influence of parents/guardians on the adolescents’ responses. All data collection was done by enumerators who were trained as per study protocols, with regular monitoring and supervision throughout the study.

Survey questionnaires consisted of 13 pre-tested modules for adolescents and one pre-tested module for parents/guardians, which were digitalized on a mobile data collection platform using CSPro version 6.3.2 (2016) on Android-based tablets. Information was collected on adolescents’ education, morbidity, dietary intake including micronutrient supplements, and physical activity. For morbidity, adolescents were asked whether they experienced at least one sign or symptom of selected health conditions in the past 14 days. Dietary intake was assessed using a 7-day food frequency questionnaire which is in line with the tool employed to estimate the Minimum Dietary Diversity for Women (available reference for assessing dietary intake at individual level) [20]. In addition, consumption of unhealthy snacks (sausage, deep fried puffy snacks, deep fried noodle snacks, and other factory-made deep fried or sugar sweetened snacks) and sugar sweetened beverages (milk shake, sweetened condensed milk, other locally-made sugary drinks and carbonated drinks) was also assessed. Physical activity patterns were assessed using 7-day activity frequency tables with a list of various sports, transport means, light exercises, household chores as well as sedentary activities, including television viewing, computer use for leisure and video game use. Respondents were asked the number of days and the average duration in hours of each activity on weekdays and the average duration in hours of each activity on weekend days in the previous week.

The parents/guardians of adolescents were requested to provide information on sociodemographic characteristics, household food security, sources of drinking water and access to sanitation facilities. Household food security was assessed using the Household Food Insecurity Access Scale (HFIAS) [21]. Drinking water sources included piped water, well with pump, protected well, protected spring, bottled water, refilled water, unprotected well, unprotected spring, river/lake/pond/irrigation/dam, rain, and other sources. Household sanitation facilities included private facilities with or without septic tank, shared/public latrine, pit latrine without slab, yard/bush/forest, river/stream/creek, and other sources.

Adolescents’ height and weight were measured following the standard procedures. Specifically, height was measured to the nearest 0.1 cm using a SECA 206 Mechanical Measuring Tape (Microtoise), and weight was recorded to the nearest 0.1 kg using a regularly calibrated SECA 874 flat digital weighing scale. Height and weight were measured and recorded twice by the same enumerator, with an acceptable difference in height measurement of 0.2 cm. Various sources such as the birth certificate, family/ID card or other official documents were used to verify the adolescent’s date of birth. If this was not possible, self-reported date of birth was recorded.

Ethical approval was obtained from the Ethical Committee of Gadjah Mada University in Yogyakarta, Indonesia (Ke/FK/0200/EC/2017). Written informed consent was obtained from all adolescents and their parents/guardians. If the parent/guardian of the respondent was illiterate, the consent form was read aloud, and a fingerprint was taken as consent to take part in the study in lieu of a signature. In addition to the ethical clearance, formal approvals were sought from the central government, provincial governments and local authorities in both districts prior to the start of the data collection activities.

### Statistical analysis

A dichotomous variable for dietary diversity was created to classify adolescents into low (<5 food groups) and high (≥5 food groups) diet diversity [20]. Physical activities were classified into vigorous-intensity (i.e. activities that require hard physical effort and cause large increases in breathing or heart rate such as pumping water, playing football) and moderate-intensity (i.e. activities that require moderate physical effort and cause small increases in breathing or heart rate such as cycling to school, sweeping the house) based on the WHO Global Recommendations on Physical Activity for Health for children aged 5–17 years [22]. Household wealth quintiles were created using the approach outlined by the Demographic Health Surveys [23]. The wealth index was developed using a principal component analysis based on 13 variables including the type of floor material, type of toilet, use of septic tank, type of cooking fuel, maternal education, use of LPG gas, ownership of assets (refrigerator, radio, bicycle, motorcycle, and car/truck), food insecurity, and monthly income. Household food security was dichotomized into food secure and food insecure (mild, moderate or severe insecurity) households. Drinking water sources were dichotomized into improved (piped water, well with pump, protected well, protected spring, bottled water, refilled water) or unimproved (unprotected well, unprotected spring, river/lake/pond/irrigation/dam, rain, and others) sources. Household sanitation facilities were dichotomized into private (private with or without septic tank) and non-private (shared/public latrine, pit latrine without slab, yard/bush/forest, river/stream/creek, and others).

Body mass index (BMI) was defined as weight (kg)/height (m^2^). Indicators of stunting (height-for-age z-score [HAZ] <-2 standard deviation [SD]), thinness (BMI-for-age z-score [BAZ] <-2 SD), overweight (BAZ >1 SD and ≤2 SD) and obese (BAZ >2 SD) were estimated using the 2007 World Health Organization (WHO) growth reference [24]. In logistic regression, overweight was defined as BAZ >1 SD.

Data entered into the digital questionnaire were double-checked by trained enumerators and supervisor. All anthropometric data were double-entered and appropriate data cleaning procedures were conducted. The analyses were weighted according to the total population size and adjusted for the multistage sampling design.

**Fig 2:**
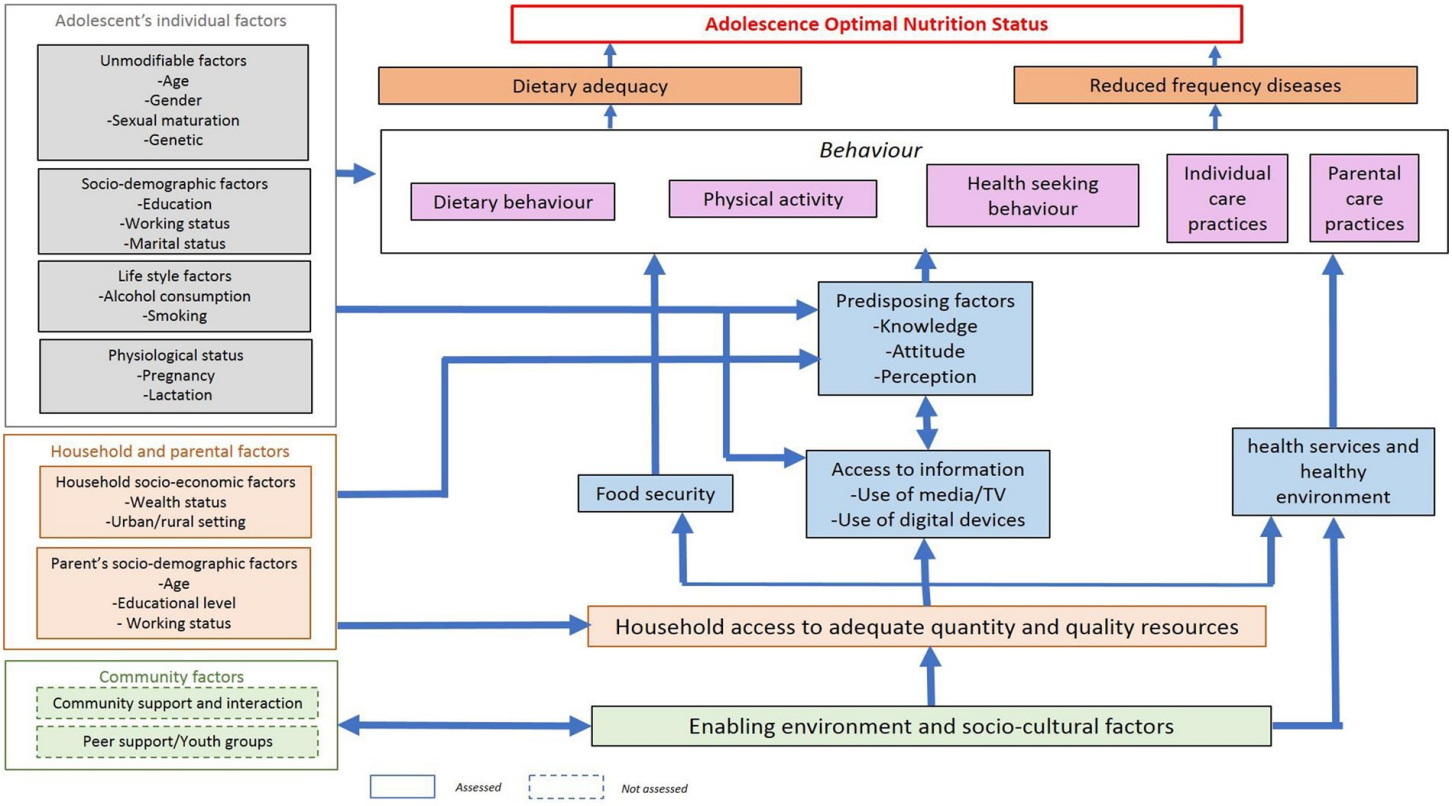
Conceptual framework of nutritional status among adolescents (UNICEF Indonesia 2017).

Descriptive analyses examined the distribution of the full range of variables. Determinants of stunting, thinness and overweight were analyzed with multivariable logistic regression using a generalized estimation equation. Separate logistic regression models were developed to examine the association between the nutritional status of interest (i.e. stunting, thinness and overweight) and hypothesized determinants. The model to assess the association between thinness and hypothesized determinants was run in comparison to normal weight individuals. Similar approach was taken for the model to determine the association between overweight and hypothesized determinants. The goodness of fit of all the regression models was assessed using chi-square tests for a generalized estimation equation. The determinant analysis was guided by the adolescent nutrition conceptual framework (Fig 2).

Variables were selected for inclusion in the multivariable models if they had a significant association with the dependent variable in the bivariable analyses (*p*<0.05). The variables explored as factors associated with adolescent nutritional status included community-level characteristics, including district and residence (urban vs. rural), the household/parental-level characteristics, and the adolescent-level characteristics, including sociodemographic status, morbidity, dietary intake, physical activity and others. Adolescent age and sex were included in the regression models irrespective of statistical significance. In multivariable models, alpha of <0.05 was considered statistically significant. All data analyses were conducted using the Stata statistical software package version 15.0 (Stata Corp., College Station, TX, USA).

## Results

Overall, survey refusals (Klaten: 6.7%; Lombok Barat: 2.7%) and drop outs (Klaten: 0.5%; Lombok Barat: 0.6%) were minimal. About half the adolescents were living in Klaten and urban areas (Table 1). The older age group (15–18 years) made up 55% of adolescents. The majority (94%) of the adolescents were enrolled in school at the time of interview, but 28% of adolescents reported having worked for cash or in kind. Nearly one in five adolescents reported having digestive problems (e.g. nausea, vomiting) in the past two weeks. Approximately two thirds (63%) of adolescents performed vigorous-intensity physical activity at least once in the previous week, while more than half (55%) reported watching television two hours or more every day in the past week. The majority of the households had improved water sources (87%) and private sanitation facility (76%).

**Table 1:** Characteristics of adolescents, their household/parents and the community included in analysis (N = 2160)*.

Characteristics	% (*n*)
**Age**	
12–14 years	45.3 (978)
15–18 years	54.7 (1182)
**School enrolment**	
Currently enrolled in school	93.8 (2027)
Currently not enrolled in school	6.2 (133)
**Highest education attainment**	
No education or incomplete primary	10.5 (225)
Complete primary or incomplete junior high	50.6 (1087)
Complete junior high or above	38.9 (836)
**Employment**	
Never worked for cash or in kind	71.8 (1550)
Ever worked for cash or in kind	28.2 (610)
**Morbidities in the past two weeks**	
Diarrhoea	8.4 (181)
High fever	2.1 (44)
Digestive problems^1^	18 (385)
**Physical activity in the past 7 days**	
Performed vigorous-intensity physical activity at least once^2^	63.4 (1369)
Performed moderate-intensity physical activity at least once^3^	98.6 (2126)
Watching television ≥2 hours everyday^4^	54.7 (1181)
Playing videogames at least once^5^	10.2 (220)
Leisure time computer use at least once^5^	20.1 (435)
**Drinking water source**	
Improved drinking water sources	86.6 (1870)
Unimproved drinking water sources	13.4 (290)
**Sanitation facility**	
Private sanitation facility	75.8 (1637)
Non-private sanitation facility	24.2 (523)
**Maternal education**	
No education or incomplete primary	29.1 (620)
Complete primary or incomplete junior high	28.5 (606)
Complete junior high or above	42.4 (904)
**Paternal education**	
No education or incomplete primary	22.9 (483)
Complete primary or incomplete junior high	25.2 (530)
Complete junior high or above	51.9 (1093)
**Residence**	
Urban	47.8 (1032)
Rural	52.2 (1128)

* Missing values existed for adolescent education attainment (*n* = 12), diarrhea (*n* = 8), high fever (*n* = 9), digestive problems (*n* = 9), moderate intensity physical activity (*n* = 3), maternal education (*n* = 30) and paternal education (*n* = 54).

^1^ Digestive problems included nausea, vomiting, bloated feeling and pain (but not diarrhea).

^2^ Vigorous-intensity physical activity included carrying heavy loads (>10kg), pushing heavy loads (>30kg), pumping water, playing football, running, jumping ropes and playing martial arts.

^3^ Moderate-intensity physical activity included climbing up and down stairs, cycling to school, cycling for leisure, walking to school, walking for leisure, playing with siblings while not sitting, taking care of younger siblings, swimming, aerobic, playing basketball, baseball, volleyball, badminton, tennis or table tennis, gymnastics, yoga, dancing, cheerleading, weightlifting and chores such as working in the garden, sweeping the house, mopping the house, washing the bathroom, cleaning one’s bedroom and taking care of livestock and the field.

^4^ Each adolescent was asked the number of days and the average duration in hours of TV viewing on week days and the average duration in hours on Saturday and Sunday in the previous week.

^5^ Percentage of adolescents who performed any sedentary activity in the past seven days.

A quarter (25%, n = 268) of girls were stunted, 5% (n = 55) thin, 8% (n = 86) overweight and 2.9% (n = 31) were obese (Table 2). Among boys, 21% (n = 229) were stunted, 11% (n = 121) thin, 8.3% (n = 90) overweight and 3.2% (n = 34) were obese. More than half (52%, n = 1132) of adolescents reported poor dietary diversity in the previous week. Only about half the adolescents consumed fruits and vegetables daily. In contrast, more than a quarter and 69% of them consumed sugar sweetened beverages and unhealthy snacks every day, respectively. Half the adolescents resided in food insecure households.

**Table 2:** Nutritional status of adolescents and nutrition-related indicators*.

	%, (*n*)	
**Nutritional status**	Girl	Boy
HAZ^1^ (mean±SD)	-1.45 (±0.84)	-1.28 (±0.99)
BAZ^1^ (mean±SD)	-0.31 (±1.1)	-0.62 (±1.24)
Stunted^2^	24.8 (268)	21.2 (229)
Thin^2^	5.1 (55)	11.2 (121)
Overweight^2^	8 (86)	8.3 (90)
Obese^2^	2.9 (31)	3.2 (34)
**Dietary intake in the past 7 days**		
Consumed <5 food groups everyday^3^	52.4 (1132)	
Consumed ≥5 food groups everyday^3^	47.6 (1027)	
Consumed protein-rich food everyday^4^	36.5 (788)	
Consumed fruits and vegetables everyday^5^	53.1 (1147)	
Consumed sugar sweetened beverages at least once everyday	33.9 (732)	
Consumed unhealthy snacks at least once everyday	68.9 (1489)	
**Household food security**^6^		
Secure	44.9 (969)	
Mildly insecure	21.5 (464)	
Moderately insecure	24.4 (527)	
Severely insecure	9.3 (200)	

* Missing values existed for stunted (*n* = 1), thin (*n* = 8), and overweight or obese (*n* = 8)

^1^ HAZ, height-for-age Z-score; BAZ, Body Mass Index (BMI)-for-age Z-score. HAZ and BAZ were calculated using the 2007 WHO growth reference.

^2^ Stunted and thin were defined as HAZ and BAZ <-2 SD, respectively. Overweight was defined as BAZ >+1 SD and ≤+2 SD. Obese was defined as BAZ >+2 SD.

^3^ 10 food groups in the food frequency questionnaire included cereal/grains/white roots/tubers/plantains, pulses/beans, nuts and seeds, dairy, meat/poultry/fish, eggs, dark green leafy vegetables, other vitamin A rich fruits and vegetables, other vegetables and other fruits.

^4^ Protein-rich food included mean, poultry, fish and eggs.

^5^ Fruits and vegetables included dark green leafy vegetables, other vitamin A rich fruits and vegetables, other vegetables and other fruits.

^6^ Assessed by Food and Nutrition Technical Assistance (FANTA). 2007. Household Food Insecurity Access Scale (HFIAS) for Measurement of Food Access: Indicator Guide. Version 3. Washington, D.C.: FANTA.

In the multivariable analysis for stunting, living in Klaten (OR = 0.63; 95% CI: 0.51–0.79) and in wealthier households (OR = 0.67; 95% CI: 0.49–0.91) was protective against stunting, while older age (OR = 1.88; 95% CI: 1.42–2.46) predicted stunting (Table 3). Higher education attainment was also associated with lower odds of being stunted (complete primary or incomplete junior high: OR = 0.65; 95% CI: 0.43–0.98, complete junior high or above: OR = 0.52; 95% CI: 0.33–0.88).

**Table 3:** Factors associated with stunting in adolescents 12–18 years in Klaten and Lombok Barat districts, Indonesia, 2017.

Characteristic	N	% stunted	Crude OR (95% CI)	*p*-value	Adjusted OR (95% CI)	*p*-value
*Community level*						
**District**						
Lombok Barat	1080	28.6	1.00		1.00	
Klaten	1079	17.4	0.53 (0.43–0.64)	<0.001	0.63 (0.51–0.79)	<0.001
*Household/parental level*						
**Wealth status**^1^						
Poorest and poorer	864	29.5	1.00		1.00	
Middle	432	23.2	0.73 (0.53–0.99)	0.043	0.90 (0.64–1.27)	0.56
Wealthier and wealthiest	863	16.5	0.48 (0.38–0.59)	<0.001	0.67 (0.49–0.91)	0.012
**Food security**^2^						
Secure	968	18.6	1.00		1.00	
Not secure	1191	26.62	1.52 (1.26–1.84)	<0.001	1.19 (0.92–1.51)	0.18
**Drinking water source**						
Unimproved	290	29.3	1.00		1.00	
Improved	1869	22	0.70 (0.53–0.94)	0.018	0.80 (0.60–1.07)	0.138
**Sanitation facility**						
Non-private	523	28.9	1.00		1.00	
Private	1636	21.2	0.70 (0.56–0.87)	0.001	0.90 (0.71–1.13)	0.357
**Maternal education**						
Incomplete primary	620	27.4	1.00		1.00	
Complete primary or incomplete junior high	606	25.4	0.91 (0.70–1.19)	0.504	1.23 (0.91–1.65)	0.173
Complete junior high or above	903	18.3	0.62 (0.50–0.77)	<0.001	1.17 (0.85–1.57)	0.294
*Adolescent level*						
**Age group**						
12–14 years	977	19.8	1.00		1.00	
15–18 years	1182	25.7	1.42 (1.18–1.70)	<0.001	1.88 (1.42–2.46)	<0.001
**Sex**						
Boy	1079	21.2	1.00		1.00	
Girl	1080	24.8	1.22 (1.00–1.51)	0.052	1.23 (0.99–1.54)	0.056
**Education attainment**						
Incomplete primary	224	29	1.00		1.00	
Complete primary or incomplete junior high	1087	21.4	0.67 (0.46–0.99)	0.045	0.65 (0.43–0.98)	0.04
Complete junior high or above	836	23.6	0.78 (0.55–1.12)	0.18	0.52 (0.33–0.88)	0.005
**Watched television in the past 7 days**						
<2hrs per day	979	25.2	1.00		1.00	
≥2hrs per day	1180	21.2	0.80 (0.66–0.99)	0.038	0.88 (0.71–1.08)	0.233

^1^ Household wealth quintile was generated using principal component analysis.

^2^ Assessed by Food and Nutrition Technical Assistance (FANTA). 2007. Household Food Insecurity Access Scale (HFIAS) for Measurement of Food Access: Indicator Guide. Version 3. Washington, D.C.: FANTA.

For thinness, girl sex was associated with 58% reduced odds of being thin (95% CI: 0.30–0.58) (Table 4). There was no association between thinness and morbidities, dietary intake or household food security.

**Table 4:** Factors associated with thinness in adolescents 12–18 years in Klaten and Lombok Barat districts, Indonesia, 2017.

Characteristic	N	% thin	Crude OR (95% CI)	*p*-value	Adjusted OR (95% CI)	*p*-value
*Adolescent level*						
**Age group**						
12–14 years	862	10	1.00		1.00	
15–18 years	1049	8.6	0.89 (0.63–1.15)	0.283	0.84 (0.62–1.13)	0.244
**Sex**						
Boy	955	12.7	1.00		1.00	
Girl	956	5.8	0.42 (0.31–0.58)	<0.001	0.42 (0.30–0.58)	<0.001

Adolescents from higher household wealth quintiles had increased odds of being overweight (middle quintile: OR = 2.03; 95% CI: 1.32–3.13, wealthier and wealthiest quintiles: OR = 1.93; 95% CI: 1.27–2.97) (Table 5). Adolescents whose mother had complete primary or incomplete junior high school education compared to no or incomplete primary education were protected from being overweight (OR = 0.60; 95% CI: 0.40–0.90). Dietary intake and physical activity were not associated with being overweight.

**Table 5:** Factors associated with overweight in adolescents 12–18 years in Klaten and Lombok Barat districts, Indonesia, 2017.

Characteristic	N	% overweight	Crude OR (95% CI)	*p*-value	Adjusted OR (95% CI)	*p*-value
*Community level*						
**District**						
Lombok Barat	975	9.6	1.00		1.00	
Klaten	1001	14.7	1.62 (1.20–2.18)	0.002	1.28 (0.88–1.90)	0.198
*Household/parental level*						
**Wealth status**^1^						
Poorest and poorer	792	7.8	1.00		1.00	
Middle	395	14.4	1.97 (1.38–2.83)	<0.001	2.03 (1.32–3.13)	0.001
Wealthier and wealthiest	789	15.5	2.12 (1.55–2.89)	<0.001	1.93 (1.27–2.97)	0.002
**Food security**^2^						
Secure	899	14	1.00		1.00	
Not secure	1077	10.7	0.75 (0.59–0.94)	0.014	1.01 (0.77–1.32)	0.962
**Maternal education**						
Incomplete primary	562	10.5	1.00		1.00	
Complete primary or incomplete junior high	554	8.8	0.81 (0.57–1.15)	0.24	0.60 (0.40–0.90)	0.016
Complete junior high or above	833	15.6	1.51 (1.11–2.05)	0.01	0.87 (0.57–1.32)	0.508
*Adolescent level*						
**Age group**						
12–14 years	891	12.9	1.00		1.00	
15–18 years	1085	11.6	0.88 (0.68–1.13)	0.31	1.07 (0.78–1.48)	0.653
**Sex**						
Boy	958	12.9	1.00		1.00	
Girl	1018	11.5	0.88 (0.67–1.15)	0.35	0.96 (0.73–1.27)	0.772
**Education attainment**						
Incomplete primary	201	8.5	1.00		1.00	
Complete primary or incomplete junior high	998	13.9	1.77 (1.06–2.92)	0.028	1.51 (0.88–2.59)	0.139
Complete junior high or above	765	10.9	1.32 (0.77–2.27)	0.317	1.04 (0.53–2.01)	0.918

^1^ Household wealth quintile was generated using principal component analysis (PCA).

^2^ Assessed by Food and Nutrition Technical Assistance (FANTA). 2007. Household Food Insecurity Access Scale (HFIAS) for Measurement of Food Access: Indicator Guide. Version 3. Washington, D.C.: FANTA.

## Discussion

This study explored patterns of the DBM among adolescents from two districts in Indonesia. Overall, the DBM continues to affect both girls and boys, with one of ten adolescents being either thin or overweight/obese. About a quarter of adolescents were stunted. Household wealth, living in a district with lower poverty and higher adolescent education were protective against stunting, but older age was associated with being stunted. While some maternal education was associated with lower odds of being overweight, household wealth predicted adolescents’ overweight. There were fewer associations with thinness, with only girl sex protecting against being thin.

Poverty remains an important unfinished agenda in Indonesia, where high levels of income inequalities persist. Observed levels of adolescent malnutrition suggest little change from levels documented in the 2013 national survey data [3], and echo similar global patterns seen in low- and middle-income countries (LMICs) [25]. Adolescents from a district with lower socioeconomic status and from poorer households as well as older adolescents were more likely to be stunted. Stunting is a manifestation of chronic undernutrition during critical periods of growth and development. Prior studies have found an increased odds of stunting among older versus younger aged adolescents [26]. This is likely due to a combination of factors including the onset of puberty, menstruation and peak height velocity and is reflective of longer-term undernutrition [7]. The association between nutritional status and the onset of menarche has been well-documented [27,28]. Evidence also shows that a greater amount of stature may be gained among those with early menarche [29,30]. Therefore, if children enter adolescence undernourished it is likely that they will enter menarche at an older age, which could further limit their linear growth potential. Adolescence is also a period of rapid growth with increased needs for calories and micronutrients [8]. Thus, higher odds of stunting among older-aged adolescents may be reflective of longer-term calorie and micronutrient deficiencies during critical periods of growth spurts, failing to reach full height potential. This suggests that with a diminishing rate of poverty reduction in Indonesia [31], those that are economically and geographically most vulnerable may be left behind undernourished.

While rapid economic growth in Indonesia and other LMICs lifted a large proportion of the population out of poverty, living environments have become increasingly obesogenic, driving the obesity epidemic [32–35]. Well-aligned with our results, an analysis from over 100 LMICs highlighted that almost all countries are facing the DBM, and that the ratio of overweight to underweight increases in line with per capita income [35]. A pooled analysis of adolescent nutrition showed a three-fold greater prevalence of overweight and obesity relative to thinness in East and Southeast Asia [36,37]. Our study found that living in a district with higher socioeconomic status and coming from wealthier households predicted overweight. Urbanization and national and household wealth are commonly cited determinants of overweight in LMICs [38]. Prior research on adolescent nutrition in the study districts found that safe, green spaces and public sports facilities to exercise are decreasing as communities develop economically, with physical activity being replaced by screen use [13]. Adolescents in our study spent a considerable amount of time being sedentary, in particular viewing TV.

Available studies have reported mixed results of the effect of maternal education on their children’s weight status [39,40]. In developed countries, maternal education seems to have protective effects on children’s BMI, due to better knowledge about health and nutrition and more financial means to access healthier diets [39–41]. In less developed societies, maternal education tends to be positively associated with children’s weight status, due to more available financial resources to access western, energy dense food and motorized vehicles and electronic gadgets and a societal norm that fatter children are healthier and wealthier [42,43]. These contrasting results may be attributed to the developmental stage of the country, which is closely linked to epidemiological transition. Adolescents in our study were less likely to be overweight if their mother had primary or some secondary education, compared to no or incomplete primary education, while higher household wealth increased the odds of overweight. Our findings merit further investigations to understand the pathways through which maternal education and other socioeconomic status impact Indonesian adolescents’ BMI.

We found no association between dietary intake and adolescents’ nutritional status, potentially due to the limitations in our dietary intake assessment based on recall. Unlike our study, an analysis of the Global School Health Survey between 2007 and 2013 among the ASEAN member countries revealed that adolescents who had fast food twice or more per week were more likely to be overweight and obese [44]. Other studies in the region also showed a positive association between the consumption of junk foods and sugar sweetened beverages and overweight [45,46]. On the other hand, consistent with our findings, a systematic review of overweight status and associated factors in 34 countries among adolescents aged 10–16 years found no association between dietary intakes and overweight [47]. Despite no association between diets and overweight, adolescents in our study reported a relative lack of dietary diversity and regular consumption of unhealthy snacks and beverages. A nutrition transition experienced in many LMICs has altered the dietary patterns, resulting in increased consumption of energy-dense, ultra-processed foods [48]. Previous studies suggest that food environments that surround adolescents in Indonesia pose a serious challenge in promoting healthy eating behaviors and nutritional status, as Indonesian adolescents are exposed to unhealthy food choices and inappropriate marketing from school canteens, street vendors and different media platforms [5,13]. Their mothers also rely heavily on readymade meals from street food stalls to feed their family as employment away from home becomes common [13].

Adolescent sex was a risk factor for thinness as girls had a 58% lower odds of being thin as compared to boys. Adolescent boys have higher caloric needs relative to girls. A prior qualitative study in the study districts also found that adolescent boys were more physically active and reported higher prevalence of smoking, compared to girls, putting boys at higher risk of thinness [13].

The study had several limitations. First, it was a cross-sectional survey and any associations cannot be interpreted as causal. Reverse causation is also possible. Second, the amounts of food consumed were not assessed, making it difficult to assess the associations between adolescent’s nutritional status and food consumption. The use of a 7-day food frequency questionnaire without the amount of food intake being assessed might also have resulted in inaccurate estimates of food consumption. Third, physical activity was not assessed using a standardized physical activity tool like the Global Physical Activity Questionnaire. Although our assessment tool was adapted from a questionnaire employed in a national survey in India, and widely pre-tested in the Indonesian context, we acknowledge the limitation of not having used a global standard questionnaire.

Evidently, the DBM affects adolescent girls and boys between 12 and 18 years old in Klaten and Lombok Barat districts of Indonesia. All forms of adolescent malnutrition must be addressed simultaneously, targeting different nutritional needs of the population and addressing various underlying determinants at community, household and individual levels. The DBM cannot be solved by the health sector alone; it needs concerted efforts from various ranges of multi-sectoral stakeholders. A combined set of nutrition specific and sensitive interventions implemented by all relevant partners will be expected to reduce the DBM in adolescents and help halt the increasing burden of nutrition-related NCDs in Indonesia.
